# Evaluating long-term outcomes of direct-acting antiviral therapy in chronic hepatitis C: A retrospective study

**DOI:** 10.1097/MD.0000000000046449

**Published:** 2025-12-12

**Authors:** Bo Ram Sung, Sang Goon Shim, Kwang Min Kim, Jung Won Lee, Jun Young Kim, Bo Kyeong Lee, Cheon Hoo Jun, Byung Soo Kwan

**Affiliations:** a Department of Internal Medicine, Samsung Changwon Hospital, Sungkyunkwan University School of Medicine, Changwon, Republic of Korea.

**Keywords:** chronic hepatitis C, direct-acting antiviral, hepatitis C virus, hepatocellular carcinoma, liver cirrhosis

## Abstract

Direct-acting antiviral (DAA) therapy is now the recommended standard for chronic hepatitis C (CHC), demonstrating remarkable efficacy. Nonetheless, there is a relative lack of information concerning the sustained, long-term consequences of DAA treatment. Therefore, this study sought to investigate the long-term impact of DAA therapy on patients with CHC. We conducted a retrospective review of CHC patients who received DAA therapy at Samsung Changwon Hospital. Patients with preexisting hepatocellular carcinoma (HCC) and those lost to follow-up were excluded. Demographic, clinical, and virological data were analyzed, focusing on DAA treatment outcomes, liver fibrosis, and the relationship with HCC development. A total of 223 patients were included: 59.5% with CHC genotype 1, 93.3% treatment-naïve, and 26.5% with liver cirrhosis (LC). Treatment included asunaprevir and daclatasvir in 46.2%, with 46.2% receiving a 12-week regimen. Sustained virological response at 12 weeks (SVR12) was achieved in 97.8%, with 5 treatment failures. Post-treatment, APRI scores < 0.7 increased (68.2% vs 87.7%, *P* < .001), as did FIB-4 scores < 3.25 (60.1% vs 80.3%, *P* < .001). During follow-up, 9 patients developed HCC, with serum sodium identified as a risk factor (odds ratio: 0.6; confidence interval: 0.41–0.88; *P* = .008). DAA treatment effectively treats CHC and reduces liver fibrosis. However, further research is warranted to better understand and predict the development of HCC.

## 1. Introduction

The hepatitis C virus (HCV) impacts roughly one out of every hundred individuals globally.^[1]^ Chronic HCV infection leads to complications, such as liver cirrhosis (LC), severe liver disease, end-stage liver disease, and hepatocellular carcinoma, which have a mortality rate of approximately 400,000 annually.^[2,3]^ Treating individuals with chronic HCV infection is essential to halt the progression towards liver cirrhosis and hepatocellular carcinoma (HCC).^[4]^

Interferon-based therapies are the primary treatments for chronic HCV infection. However, these regimens have limited efficacy and significant adverse effects, restricting their widespread use.^[5–7]^ The development of direct-acting antiviral agents (DAAs) has fundamentally altered the HCV treatment paradigm, positioning DAA-based therapies as the primary standard of care.^[8]^ This shift occurred because of the superior efficacy of DAAs, with high virological response and cure rates coupled with improved tolerability compared with interferon-based regimens.^[9,10]^ Consequently, DAAs have transformed the landscape of chronic HCV management, offering patients more effective and better tolerated treatment options. Combination therapies with 2 to 3 DAAs have demonstrated efficacy against all types of HCV infections, with a viral response rate of over 95%. The treatment duration ranged from 8 to 24 weeks, depending on the patient’s characteristics. Since the introduction of DAAs, several studies have indicated a reduction in end-stage liver disease and, ultimately, a decrease in mortality. However, long-term data regarding the efficacy of DAA treatment remain limited compared with those of interferon-based therapies.^[11]^

Given the critical importance of long-term treatment outcomes in guiding future therapeutic decisions, we carried out a retrospective review to explore the long-term effects of DAA therapy on patients diagnosed with chronic hepatitis C (CHC).

## 2. Materials and methods

### 2.1. Study design and setting

This study was designed as a single-center, retrospective cohort analysis to assess the efficacy, safety, and long-term outcomes of DAA therapy in patients with chronic HCV infection. We collected demographic data from patients diagnosed with chronic HCV infection who received DAA treatment at Samsung Changwon Hospital, between January 1, 2016, and December 31, 2021. Patients eligible for inclusion were adults (≥18 years) with confirmed CHC who underwent DAA therapy. Our exclusion criteria encompassed those with preexisting HCC, subjects lost to follow-up, and pregnant women. The study group was observed through regular outpatient visits over a period of no <36 months.

To evaluate the efficacy of the DAA treatment, we conducted a comprehensive analysis of various clinical and laboratory parameters in all patients before and after therapy. We determined the effectiveness of DAA therapy by using sustained virological response at 12 weeks after treatment completion (SVR12) as our main outcome measure. Achievement of SVR12 was determined by the absence of measurable HCV-RNA in serum tests conducted 12 weeks post-completion of DAA therapy. For the long-term outcome assessment, we monitored the patients throughout the follow-up period and evaluated the incidence of HCC, changes in liver fibrosis markers, and various other clinical indicators as secondary endpoints.

Indications, drug selection, and treatment duration for DAA therapy for HCV treatment adhered to the guidelines established by the Korean Association for the Study of the Liver.^[12]^ The study protocol adhered to the ethical guidelines of the Declaration of Helsinki and was approved by the Institutional Review Board of Samsung Changwon Hospital, Sungkyunkwan University School of Medicine (approval no: SCMC-09-004; date: September 4, 2022). The requirement for informed consent was waived due to the retrospective nature of the study.

### 2.2. Data collection

We extracted patient demographic information and clinical parameters from electronic medical records, including age, sex, body mass index, HCV infection route, alcohol consumption, smoking, prior interferon (INF) experience, diabetes mellitus (DM), presence of chronic kidney disease (CKD), hemodialysis status, presence of LC, presence of ascites, presence of encephalopathy, HCV genotype, treated DAA regimens, and duration of treatment. The laboratory data were also collected before and after treatment including HCV-RNA, hemoglobin, platelet, aspartate aminotransferase (AST), alanine aminotransferase (ALT), alkaline phosphatase (ALP), γ-glutamyl transpeptidase (γ-GTP), total bilirubin (TB), albumin, creatinine, serum sodium, prothrombin time international normalized ratio (PT-INR), α-fetoprotein (AFP).

Furthermore, we evaluated various markers of cirrhosis and fibrosis, including the Child-Turcotte-Pugh (CTP) score, Model for End-stage Liver Disease (MELD) score, aspartate aminotransferase to Platelet Ratio (APRI) index, Fibrosis-4 (FIB-4) index, and liver stiffness measurement (LSM). LC was diagnosed on the basis of clinical assessment and imaging studies. The diagnosis of HCC was established through advanced imaging techniques, specifically computed tomography (CT) and magnetic resonance imaging (MRI). Specifically, HCC was defined as the presence of characteristic findings on either dynamic contrast-enhanced CT or dynamic contrast-enhanced MRI consistent with the diagnosis of HCC. LSMs were obtained using vibration-controlled transient elastography (FibroScan 502 touch device). MELD score, APRI index, and FIB-4 index were calculated.


$$\begin{array}{l} \begin{array}{lll} & MELD:9.57\times log_{e}\left(creatinine\right)+3.78\times log_{e}\left(bilirubin\right)\; & +11.20\times log_{e}\left(INR\right)+6.43\; \end{array} \end{array}$$

$$\begin{array}{lll} & FIB\text{-}4\;index:\;age\left(years\right)\times AST\left(U/L\right)/[PLT\left(10^{9}/L\right)\; & \times ALT^{1/2}\left(U/L\right]) \end{array}$$

$$\begin{array}{lll} & APRI=\left(AST/upper\;limit\;of\;normal\;AST\right)/PLT\;\; & \left(10^{9}/L\right)\times 100 \end{array}$$

Additionally, we investigated the adverse events related to DAA treatment. These events were categorized into 2 groups based on the Common Terminology Criteria for Adverse Events: grades 1 and 2 events were classified as mild or moderate, whereas grades 3 and 4 events were considered serious or life-threatening.

### 2.3. Statistical analysis

We summarized patients’ baseline characteristics using descriptive statistics. For categorical variables, we reported frequencies and percentages and employed Fisher exact test or Pearson chi-square test for comparisons between groups, as appropriate. Continuous variables were reported as medians with interquartile ranges. Comparisons between groups were conducted using the paired t-test for normally distributed data and the Wilcoxon signed-rank test for non-normally distributed data. The effects of treatment on liver fibrosis before and after therapy were compared using the Wilcoxon signed-rank test and are illustrated graphically. Adverse events and complications that occurred after treatment with direct-acting antiviral agents were investigated and documented.

To identify the risk factors for HCC development in patients treated with DAA agents, we conducted univariate and multivariate logistic regression analyses. Statistical significance was set at *P* value of <.05 for all analyses. All statistical analyses were conducted using Stata/SE version 16 software (StataCorp, College Station, Texas).

## 3. Result

### 3.1. Baseline characteristics

**Figure 1.**
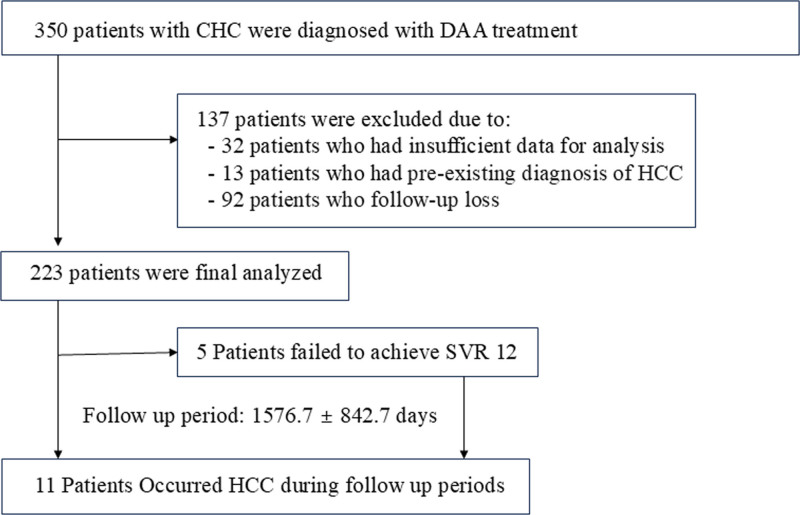
Flow chart of the patient selection process. CHC = chronic hepatitis C, DAA = direct-acting antivirals, Samsung Changwon = Samsung Changwon Hospital, Sungkyunkwan University School of Medicine, HCC = hepatocellular carcinoma, SVR 12 = sustained virologic response at 12 weeks.

Our research initially encompassed 350 chronic HCV infection cases, but the final analysis focused on 223 patients who met our inclusion criteria (Fig. 1). The mean follow-up period was 1576.7 ± 842.7 days. On average, participants in our study were 64.8 years old, with females constituting 46.5% of the group. Of the included patients, 93.3% were HCV treatment-naïve and 77.1% had an unknown HCV infection route. Additionally, 29.2% of patients had DM and 2.3% had CKD requiring hemodialysis. Additionally, 59 patients (26.5%) had LC; among these, 5 presented with mild ascites, and none exhibited hepatic encephalopathy. HCV genotype 1 was the most prevalent, accounting for 59.5% of the cases, followed by genotypes 2 (39.2 %) and 3 (1.4 %).

The DAA regimens used for treatment included asunaprevir + daclatasvir in 46.2% of patients, sofosbuvir monotherapy in 39.5%, and other regimens such as ombitasvir/paritaprevir/ritonavir + dasabuvir in 5.4% of cases. The treatment duration for patients ranged from 8 to 24 weeks, with the majority (46.6%) receiving a 12-week regimen. The initial clinical features of the study cohort are presented in Table 1.

**Table 1:** Baseline clinical characteristics of study population.

Variable	Total (N = 223)
Age, yr	64.8 ± 11.5
Sex, female	106 (47.5)
BMI, kg/m²	23.8 ± 3.3
Infection route	
Intravenous	9 (4.0)
Transfusion	2 (0.9)
Others*	40 (17.9)
Unknown	172 (77.1)
Alcohol, yes	87 (39.0)
Smoking, yes	62 (27.8)
DM, yes	65 (29.2)
Hemodialysis, yes	5 (2.3)
Presence of LC, yes	59 (26.5)
Presence of ascites	
Absent	218 (97.8)
Mild	5 (2.2)
Presence of encephalopathy, none	223 (100.0)
Genotype	
Type 1	132 (59.5)
Type 2	87 (39.2)
Type 3	3 (1.4)
Prior INF experienced	15 (6.7)
DAA regimen	
Asunaprevir + Daclatasvir	103 (46.2)
Asunaprevir + Daclatasvir + Ribavirin	4 (1.8)
Daclatasvir + Sofosbuvir	8 (3.6)
Sofosbuvir + Ribavirin	88 (39.5)
Ombitasvir/Paritaprevir/ritonavir + Dasabuvir	12 (5.4)
Grazoprevir + Elbasvir	6 (2.7)
Ledipasvir + Sofosbuvir	2 (0.9)
Duration of DAA treatment	
8 wk	2 (0.9)
10 wk	1 (0.5)
12 wk	104 (46.6)
16 wk	16 (7.2)
24 wk	100 (44.8)
Mean follow up, days	1576.7 ± 842.7

Values are presented median ± ranges or n (%).

BMI = body mass index, DAA = direct acting antiviral agent, DM = diabetes mellitus, INF = interferon, LC = liver cirrhosis, N = number of patients.

* Others included causes other than intravenous and blood transfusions, such as tattoos, drugs, and surgery.

### 3.2. Effectiveness of DAA treatment

Of 223 patients treated with DAAs, 97.8% achieved an SVR of 12. Significant changes were observed in various clinical variables following DAA treatment. HCV-RNA levels decreased (154,000 vs 15, *P *= .021), AST levels decreased (51.5 vs 24.4, *P* < .001), ALT levels decreased (51.1 vs 19.2, *P* < .001), ALP levels decreased (81.8 vs 75.8, *P* = .035), and γ-GTP levels decreased (60.9 vs 26.6, *P *< .001). Meanwhile, platelet count increased (182.1 vs 194.6, *P* < .001) and serum albumin levels also increased (4.2 vs 4.4, *P* < .001).

**Figure 2.**
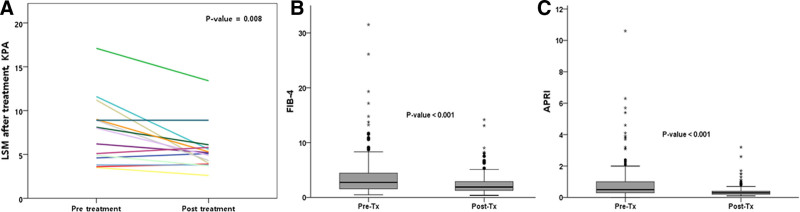
Clinical changes in LSM, FIB-4 index, and APRI index in patients following direct-acting antiviral (DAA) therapy. (a) Liver stiffness measurement (LSM) decreased following treatment with DAA (8.5 kpa vs 5.1 kpa, *P* = .008). (b) Comparison of FIB-4 index changes in patients before and after DAA treatment (39.9% vs 19.7%, *P* < .001). (c) Changes in the APRI index before and after DAA treatment (31.8% vs 12.3%, *P* < .001).

Furthermore, the proportion of patients with CTP score of 5 to 6 points increased from 94.7% before treatment to 98.7% after treatment. Concurrently, the proportion of patients with CTP score of 7 to 9 points decreased from 5.3% to 1.3%, indicating an overall improvement in liver function in patients with LC. Moreover, the proportion of patients with an APRI index ≥ 0.7 reduced (31.8% vs 12.3%, *P* < .001), FIB-4 index ≥ 3.25 reduced (39.9% vs 19.7%, *P* < .001), and LSM decreased (8.5 vs 5.1, *P* = .008). Table 2 and Figure 2 show the clinical changes observed before and after DAA treatment.

**Table 2:** Clinical characteristics before and after DAA treatment.

Variable	Total (N = 223)	Pretreatment	Post-treatment	*P*-value
HCV-RNA, IU/mL	869,000 (175,000–2640,000)	154,000 (748,000–622,000)	15 (15–120,000)	.021
Hemoglobin, g/dL	13.6 ± 1.7	13.6 ± 1.7	13.9 ± 8.4	.554
Platelet, 10^3^/μL	182.1 ± 69.6	182.1 ± 69.6	194.6 ± 71.5	<.001
AST, IU/L	51.5 ± 42.4	51.5 ± 42.4	24.4 ± 9.3	<.001
ALT, IU/L	51.1 ± 66.5	51.1 ± 33.5	19.23 ± 10.0	<.001
ALP, IU/L	81.8 ± 33.9	81.8 ± 33.9	75.8 ± 46.6	.035
γ-GTP, IU/L	58.3 ± 78.1	60.9 ± 82.8	26.6 ± 22.5	<.001
TB, mg/dL	0.8 ± 0.7	0.8 ± 0.7	0.7 ± 0.5	.422
Albumin	4.2 ± 0.5	4.2 ± 0.5	4.4 ± 0.4	<.001
Creatinine	1.0 ± 1.1	1.0 ± 1.1	1.0 ± 1.0	.808
Sodium	139.9 ± 2.8	139.7 ± 2.8	139.9 ± 2.7	.358
PT-INR	1.1 ± 0.2	1.1 ± 0.2	1.1 ± 0.1	.803
AFP, ng/mL	44.5 ± 456.3	47.5 ± 476.0	6.4 ± 25.6	.248
Ascites, mild	5 (2.2)	5 (2.2)	6 (2.7)	.317
CTP score				.014
5–6	184 (95.8)	143 (94.7)	149 (98.7)	
7–9	8 (4.2)	8 (5.3)	2 (1.3)	
MELD score				.537
<10	149 (81.4)	107 (78.1)	106 (77.4)	
10–19	28 (15.3)	24 (17.5)	22 (16.1)	
20–29	6 (3.3)	6 (4.4)	9 (6.6)	
APRI index				<.001
<0.7	150 (67.6)	150 (68.2)	193 (87.7)	
≥0.7	72 (32.4)	70 (31.8)	27 (12.3)	
FIB-4 index				<.001
<3.25	131 (59.8)	131 (60.1)	175 (80.3)	
≥3.25	88 (40.2)	87 (39.9)	43 (19.7)	
LSM, kpa	5.7 (3.8–8.9)	8.0 (4.6–9.0)	5.1 (3.9–5.8)	.008
LSM, F0-F4*				.014
F0-F1	10 (55.6)	7 (46.7)	13 (86.7)	
F2-F4	8 (44.4)	8 (53.3)	2 (13.3)	
Adverse event	33 (14.9)			
Successful SVR 12	218 (97.8)			
HCC after DAA	11 (4.9)			

Values are presented Median ± ranges or n (%).

AFP = α-fetoprotein, ALP = alkaline phosphatase, ALT = alanine aminotransferase, APRI = Aspartate aminotransferase to Platelet Ratio Index, AST = aspartate aminotransferase, CTP = Child-Turcotte-Pugh, FIB-4 = Fibrosis-4, HCC = Hepatocellular carcinoma, HCV-RNA = hepatitis C virus ribonucleic acid, LSM = Liver Stiffness Measurement, MELD = Model for End-stage Liver Disease, N = number of patients, PT-INR = prothrombin time international normalized ratio, SVR 12 = sustained virologic response 12 weeks, TB = total bilirubin, γ-GTP = γ-glutamyl transpeptidase.

* Stage of fibrosis based on LSM by using transient elastography: F1 indicates initial fibrosis, F2 indicates intermediate fibrosis, F3 indicates advanced fibrosis,and F4 indicates liver cirrhosis.

Five patients failed to achieve SVR12. Among them, 3 patients received asunaprevir + daclatasvir combination therapy, while 2 were treated with sofosbuvir monotherapy. Three of these patients had HCV genotype 1 and 2 had genotype 2. Three patients had LC, and none had received prior interferon therapy.

### 3.3. Safety of DAA treatment

Of 223 patients who received DAA treatment, 33 (14.9%) experienced adverse events. During therapy, patients experienced neither fatalities nor critical (Grade 3–4) adverse events. Some patients experienced multiple adverse events concurrently. The most frequently reported adverse event was gastrointestinal disorders, which affected 10 patients (4.5%). This was followed by hematological adverse events in 9 patients (4.0%), skin rashes in 6 patients (2.7%), and dizziness in 5 patients (2.2%).Table 3 Adverse events resulting from DAA treatment.

**Table 3:** Adverse events resulting from DAA treatment.

Variable	Total (N = 223)	Grade 1	Grade 2
Any AE	33 (14.9)	31	2
Discontinuation	0 (0.0)		
Death	0 (0.0)		
Specific AE			
Fatigue	3	3	
Dizziness	5	5	
Headache	2	2	
Dyspnea	1	1	
Cardiomegaly	1	1	
Cough	1	1	
Sore throat	1	1	
GI upset			
Dyspepsia	7	7	
Constipation	1	1	
Diarrhea	2	2	
Skin rash	6	5	1
Hematologic			
Anemia	6	5	1
Neutropenia	1	1	
Thrombocytopenia	2	1	1

Values are presented Median ± ranges or n (%).

AE = adverse event, GI = gastrointestinal, N = number of patients.

### 3.4. Long-term follow up and HCC incidence related risk factors

During the follow-up period, 11 of the 223 patients developed HCC after DAA treatment. Among the patients who developed HCC, 11 achieved SVR 12. Six of these patients had HCV genotype 1, and 5 had genotype 2. Nine out of the 11 patients had LC.

Univariate analysis identified several risk factors for hepatocellular carcinoma (HCC). These included the presence of LC (odds ratio [OR], 14.58; 95% confidence interval [CI], 3.05–69.71; *P* = .001), PT-INR (OR, 1.48; 95% CI 1.14–1.92, *P* = .004), serum sodium levels (OR; 0.69, 95% CI 0.55–0.86, *P* = .001), Platelet counts (OR; 0.97, 95% CI 0.96–0.98, *P* < .001), MELD score 10-19 (OR; 29.40, 95% CI 5.83-148.32, *P* < .001), and FIB-4 index ≥ 3.25 (OR 7.35, 95% CI 1.55–34.88), *P* = .012). In the multivariable analysis, serum sodium level emerged as a statistically significant predictor (OR, 0.60; 95% CI, 0.41 to 0.88; *P* = .008). Table 4 presents the analysis of the risk factors for HCC.

**Table 4:** Multivariable analysis of risk factors for the occurrence of HCC.

	Univariable		Multivariable	
	OR (95% CI)	*P*-value	OR (95% CI)	*P*-value
Age, yr	1.06 (0.99–1.12)	.074		
Sex, male	2.52 (0.65–9.76)	.181		
BMI, kg/m^2^	0.97 (0.79–1.18)	.747		
Cigarette smoking, yes	1.52 (0.43–5.38)	.518		
Alcohol intake, yes	1.32 (0.39–4.47)	.654		
Prior INF experienced	1.41 (0.17–1.85)	.749		
DM	0.91 (0.23–3.53)	.888		
PT-INR	1.48 (1.14–1.92)	.004	1.37 (0.83–2.25)	.221
Platelet	0.97 (0.96–0.98)	<.001	0.98 (0.96–1.01)	.150
Sodium	0.69 (0.55–0.86)	.001	0.60 (0.41–0.88)	.008
Presence of LC	14.58 (3.05–69.71)	.001	3.90 (0.42–36.18)	.232
MELD score				
<10	Reference			
10–19	29.40 (5.83–148.32)	<.001	3.15 (0.37–27.07)	.296
CTP score				
5–6	Reference			
7–9	2.78 (0.31–25.06)	.363		
≥10				
FIB-4 index				
≥3.25	7.35 (1.55–34.88)	.012	0.46 (0.02–10.58)	.625
APRI score				
≥0.7	2.64 (0.78–8.95)	.120		
AFP	0.99 (0.99–1.00)	.868		
Successful SVR 12, none	5.20 (0.53–50.91)	.157		
Adverse event	1.28 (0.26–6.22)	.757		

AFP = α-fetoprotein, APRI = Aspartate aminotransferase to Platelet Ratio Index, BMI = body mass index, CTP = Child-Turcotte-Pugh, DM = Diabetes mellitus, FIB-4 = Fibrosis-4, HCC = Hepatocellular carcinoma, INF = interferon, LC = Liver cirrhosis, MELD = Model for End-stage Liver Disease, N = number of patients, PT-INR = prothrombin time international normalized ratio, SVR 12 = sustained virologic response 12 weeks.

## 4. Discussion

Our findings indicate that DAA treatment is highly effective and well-tolerated for chronic HCV infection in South Korean patients. With 97.8% of patients achieving SVR 12, the results align with those of previous clinical trials and real-world studies, confirming that DAAs are a cornerstone for HCV eradication.^[8]^ The study also revealed significant improvements in liver function and fibrosis markers post-treatment, including reductions in AST, ALT, and γ-GTP levels, and improvements in APRI and FIB-4 scores.^[13,14]^ Safety outcomes were encouraging, with adverse effects observed in only 14.83% of the patients and no serious events requiring treatment discontinuation. This corroborates the well-established tolerability of DAAs.^[15,16]^

As a result of the long-term follow-up observations, the development of HCC in 11 post-treatment patients highlights the need for continued vigilance in monitoring patients after achieving SVR 12. Our study identified serum sodium level as a significant risk factor for HCC development, consistent with previous research demonstrating an association between hyponatremia, advanced liver cirrhosis, and HCC occurrence.^[17–20]^ This finding highlights the critical importance of careful monitoring of serum sodium levels during follow-up after CHC treatment. However, in our multivariate analysis of the factors associated with HCC occurrence, LC did not emerge as a statistically significant predictor. We posit that this unexpected finding may be attributed to the composition of our study cohort, which included only 59 patients with LC, of whom 9 developed HCC. Furthermore, most patients with cirrhosis have compensated for their diseases. The limited sample size and predominance of compensated LC may have affected the statistical power to detect a well-established association between cirrhosis and HCC risk.

While the findings of this study are consistent with the existing literature on DAA efficacy and safety, its focus on real-world outcomes in the South Korean population contributes valuable data to the global understanding of DAA effectiveness. Moreover, given the paucity of long-term follow-up data after DAA treatment, our study offers important insights into posttreatment outcomes. Long-term data on the occurrence of HCC following DAA treatment are of paramount importance. Although some studies have explored the association between DAA treatment and HCC, the relationship between these 2 factors requires further investigation.^[21–23]^

The interpretation of our results should take into account several restricting factors, including the study’s retrospective nature, its focus on a single medical center, and the relatively modest size of the patient group examined. Furthermore, this study did not include recently introduced pan-genotypic DAAs as they were not registered at the time of data collection and lacked long-term follow-up data. Future studies should focus on the long-term outcomes of these new pan-genotypic DAA regimens, as they are becoming increasingly prevalent in clinical practice.

In conclusion, our research substantiates the superior effectiveness of DAA regimens for chronic hepatitis C treatment. We found notable advancements in liver functionality and a clear decrease in hepatic fibrosis in patients who underwent DAA therapy. Importantly, our results highlight the critical need for careful monitoring of serum sodium levels during posttreatment follow-up, as hyponatremia has emerged as a significant risk factor for the development of HCC. Despite the high cure rates achieved with DAA therapy, the risk of HCC persists, even after successful viral eradication. This highlights the importance of continued vigilance and active surveillance in all patients who have undergone CHC treatment, regardless of their cirrhosis status or achievement of a sustained virological response. Our study provides valuable real-world data to the growing body of evidence supporting the use of DAAs in CHC management. This emphasizes the need for long-term follow-up studies to better understand and mitigate the residual risk of HCC in this patient population.

## Acknowledgments

The authors acknowledge the support provided By Samsung Changwon Hospital for facilitating data collection and analysis. They also thank the medical staff of the Division of Gastroenterology, Samsung Changwon Hospital, for their assistance in data collection and patient management.

## Author contributions

**Conceptualization:** Sang Goon Shim.

**Data curation:** Cheon Hoo Jun.

**Formal analysis:** Bo Kyeong Lee.

**Resources:** Cheon Hoo Jun.

**Supervision:** Byung Soo Kwan.

**Writing – review & editing:** Kwang Min Kim, Jung Won Lee, Jun Young Kim.

**Writing – original draft:** Bo Ram Sung.
